# Commentary to Single‐Cell Transcriptomics Reveal Human Skin Pathways and Introduction of a New Dior Skin Longevity Compass™

**DOI:** 10.1111/jocd.70808

**Published:** 2026-04-07

**Authors:** Michael H. Gold

**Affiliations:** ^1^ Gold Skin Care Center Nashville Tennessee USA; ^2^ Tennessee Clinical Research Center Nashville Tennessee USA

For many years now, considerable scientific advances have been made in the field of longevity science and reverse aging. Important milestones were the Lopez‐Otin article (in 2013, 2023) and Kroemer et al. (in 2025), which describe the hallmarks of aging at the organismal level. Regarding the skin, several literature reviews have been conducted, but few studies allow for a precise understanding of the hallmarks of aging at the skin level. It is therefore with particular interest that I read the Editorial Submission to the Journal of Cosmetic Dermatology, *Single‐Cell Transcriptomics Reveal Human Skin Pathways*, by Kseniya Melyukhina, Anne‐Laure Bulteau, Carine Nizard, Karl Pays, Jesse Poganik R, Vadim Gladyshev N, and Laure Crabbe. This fascinating paper supported by LVMH Recherche for Dior introduces us to potential new understandings and findings in what is typically described as the Hallmarks of Aging applied to skin.

Skin aging, as we are learning, can be associated with concurrent systemic aging. As we advance the science of skin aging, we are now able to focus on the cellular level in ways we have never been able to do in the past. And by doing so, we can develop new therapeutic options which, as of now, seem primed to reverse skin aging at the source. With this ability now coming to fruition, we envision that through reversing skin aging, we also have pathways set in place for the skin to be part of the guidance for systemic systems to be improved as well, all leading to generalized better health and longevity.

Indeed, it has been observed that many of the pathways elucidated in skin aging are common with the hallmarks of aging described by López‐Otín et al. in Cell in 2023, suggesting that human skin follows a similar aging path as other organs [1].

Longevity and regenerative medicine are terms that have been around in medicine and dermatology for a long time. For many dermatologists, these terms were conceptual in nature, and the science behind them was sorely lacking. Over the past several years, as we have delved further into the hallmarks of aging, we have begun to understand more about how the skin ages and what is required to develop meaningful therapies that can reverse the signs and symptoms for many.

The authors of this article have expanded knowledge on the hallmarks of skin aging at a cellular level. This is not a simple task. Drawing on a large study of over 300 000 human cells, carried out on a group of young donors and a second group of older donors –for us to see what these differences might look like—they performed single‐cell transcriptomics and bioinformatic analyses on fibroblasts (FB) and epidermal keratinocytes (KT). More than 12 000 genes were scrutinized, and by leveraging a tool combining statistical algorithms with artificial intelligence capabilities, they have sifted through more than 1000 biomarkers, allowing for the objective identification of the biological processes affected by age within both keratinocytes and fibroblasts.

Both FB and KT showed strong up‐regulation of genes associated with inflammation, and a down‐regulation of genes associated with oxidative stress detoxification. As well, the analysis revealed an imbalance in the expression of circadian genes and an activation of autophagy with an upregulation of mTOR.

In this evaluation, aged FB expressed higher levels of senescence markers as compared to younger FB as well as decreased expressions of epidermal extra cellular matrix components. The FB is vitally important for the production of the extra cellular matrix. The researchers also found that the gene regulation responsible for this production of this extra cellular matrix had a significant impact with aging, another important finding that Kroemer and al. also described in their revised version of the hallmarks of organismal aging published in 2025. This study therefore brought to light three new features of skin aging in addition to the 12 previously identified [2].

These findings are all instrumental in skin aging and will add to our scientific knowledge of longevity and regenerative medicine. The hallmarks of aging as we know them have changed over time, and with new outcomes described within this article, it is time to consider changing the hallmarks once again to adapt them to skin.

This discovery, combined with many years of research in the field, has given rise to the new Dior Skin Longevity Compass™: a unique tool describing the 15 hallmarks of skin aging and guiding us, like a compass, towards solutions to visibly reverse the effects of aging.

This new approach is distinguished by:

Its perfect application to the skin: Far from wheels that apply to the body rather than the skin specifically, this new Dior Skin Longevity Compass™ is the result of the analysis of human skin cells and thus focuses on how to reverse aging at the skin level.

Its extreme precision: Beyond investigations made on the entire skin tissue without any distinction, this new study, analyzing more than 1000 cellular biomarkers on both keratinocytes and fibroblasts, allows for the deciphering of the pathways to reverse the effects of skin aging.

Its completeness: Beyond highlighting new important pathways, this approach enables us to understand more precisely the main pathways of skin aging.

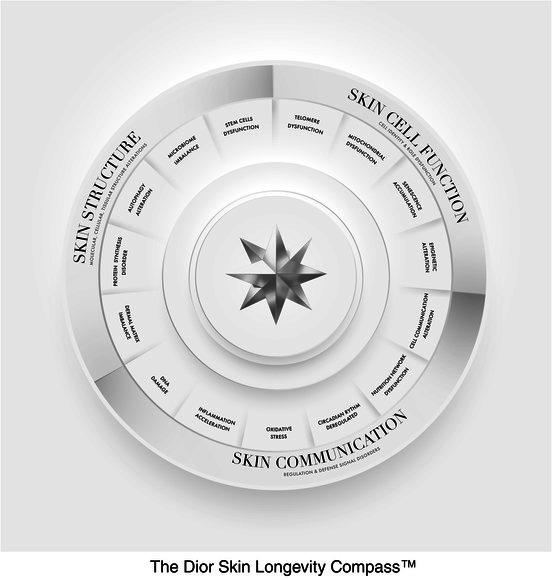

## Conflicts of Interest

The author declares no conflicts of interest.

## Data Availability

The data that support the findings of this study are available from the corresponding author upon reasonable request.
